# Experimental observation of the improvement in MTF from backthinning a CMOS direct electron detector

**DOI:** 10.1016/j.ultramic.2009.05.005

**Published:** 2009-08

**Authors:** G. McMullan, A.R. Faruqi, R. Henderson, N. Guerrini, R. Turchetta, A. Jacobs, G. van Hoften

**Affiliations:** a MRC Laboratory of Molecular Biology, Hills Road, Cambridge CB2 0QH, UK; b STFC Rutherford Appleton Laboratory, Chilton, Didcot OX11 0QX, UK; c Technical University of Eindhoven, 5600 MB Eindhoven, Netherlands; d FEI Electron Optics, 5600 MD Eindhoven, Netherlands

**Keywords:** CMOS detectors, Backthinning, MTF, Electron microscopy, Pixel detectors

## Abstract

The advantages of backthinning monolithic active pixel sensors (MAPS) based on complementary metal oxide semiconductor (CMOS) direct electron detectors for electron microscopy have been discussed previously; they include better spatial resolution (modulation transfer function or MTF) and efficiency at all spatial frequencies (detective quantum efficiency or DQE). It was suggested that a ‘thin’ CMOS detector would have the most outstanding properties [1–3] because of a reduction in the proportion of backscattered electrons. In this paper we show, theoretically (using Monte Carlo simulations of electron trajectories) and experimentally that this is indeed the case.

The modulation transfer functions of prototype backthinned CMOS direct electron detectors have been measured at 300 keV. At zero spatial frequency, in non-backthinned 700-μm-thick detectors, the backscattered component makes up over 40% of the total signal but, by backthinning to 100, 50 or 35 μm, this can be reduced to 25%, 15% and 10%, respectively. For the 35 μm backthinned detector, this reduction in backscatter increases the MTF by 40% for spatial frequencies between 0.1 and 1.0 Nyquist. As discussed in the main text, reducing backscattering in backthinned detectors should also improve DQE.

## 1 Introduction

Electronic detectors are becoming increasingly attractive for a range of applications in electron microscopy as discussed in a number of recent publications [2,4–6]. Imaging on film, the traditional medium for recording in electron microscopy, is slow and tedious requiring film development and densitometry before results are available. A great attraction of electronic detectors is that the user has immediate feedback in the form of images, which can be processed and stored for further off-line analysis. Over the last decade, indirect electron detectors (i.e. using a phosphor to convert the electron energy into light) have already replaced film for recording images in many types of less demanding applications in electron microscopy [7,8]. These are predominantly CCD detectors with a phosphor/fibre-optics front end and, whilst these detectors have an excellent signal-to-noise ratio at low resolution, they invariably have poor spatial resolution (low modulation transfer function (MTF) at high spatial frequencies). This limitation does not affect applications where the specimen is not radiation sensitive, since the poor detector performance can be compensated simply by increasing the electron dose as in many materials science applications [9]. However, when the specimen being imaged can only tolerate a limited dose before damage sets in, there is a prime requirement for a fast, efficient detector with a good MTF. This includes biological electron tomography [10], single particle electron cryomicroscopy [11] and electron crystallography of organic or biological molecules [12]. The need for a high MTF means that the contribution of any backscattered electrons to the observed signal must be minimised, as discussed in more detail below. It has been shown recently that even backscattering from the plastic support in film significantly reduces the MTF of film at high resolution [13]. With this goal, we have measured the improvement in MTF on a number of prototype complementary metal oxide semiconductor (CMOS) detectors that have been backthinned to different thicknesses. The improvement is in agreement with Monte Carlo simulations of electron trajectories in silicon and suggests that backthinning will be important in all future detectors for electron microscopy.

## 2 Detector

The general requirements of detectors for electron microscopy have been reviewed [5] and a more detailed paper on the measurement of detective quantum efficiency (DQE, and DQE(*ω*), where *ω* represents spatial frequency) of CMOS and other detectors has been published recently [13]. It was pointed out that CMOS detectors had the potential for providing excellent MTF and DQE at 300 keV provided backscattering from the substrate was eliminated or reduced substantially. A key property of detectors, which is vital in obtaining images with high resolution is the point spread function, which is directly related to the modulation transfer function. Two components which affect MTF are: diffusion effects in the epilayer leading to a deterministic blur but which does not degrade DQE; backscattering from the substrate which does degrade DQE. Backthinning reduces the second component, viz. backscattering so it should also improve DQE [14].

**Fig. 1:**
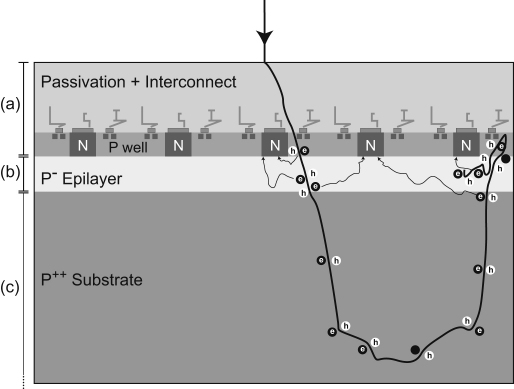
Schematic of MAPS detector shown in cross-section. The detector has three main regions: (a) about 5-μm-thick passivation layer plus interconnections for readout electronics in the P well, (b) a few microns of lightly doped epilayer where the useful signal is generated, and drifts on to N wells prior to being read out, and (c) the main bulk of the detector, the substrate, which is heavily doped and which does not play a significant role in the detection process. A possible path for a single incident high-energy electron is shown to illustrate the problem with backscatter from the substrate.

Images recorded by direct electron incidence on CMOS detectors potentially have contributions from two components. The first contribution arises from the energy deposited due to the initial passage of the electron through the epilayer and the second from the subsequent energy deposited after backscattering from the substrate. An example of backscattering is illustrated in Fig. 1, which shows the pixel layout of a typical CMOS sensor in cross-section. The track of a single electron is shown, derived from a Monte Carlo simulation with the incident energy chosen to be 300 keV. For the present purposes the cross-section of the pixel can be divided into three regions. The top layer through which the electron enters the pixel, marked (a) in the figure, consists of the passivation layer and interconnects. This is followed by the lightly doped p^−^epilayer, marked as (b), containing the n-well diodes and the p^−^ wells where the in-pixel transistors sit. The last element in each pixel is the heavily doped p^++^ substrate, marked as (c), which plays a very minor role in the signal generation. The dimensions of the three regions are typically a few microns for the passivation layer, up to 20 μm for the epilayer and a few hundred microns for the substrate [15].

The passage of an electron creates electron-hole pairs in all parts of the sensor but only the electrons created in the epilayer, region (b) have a sufficiently long lifetime to diffuse to the nearest n-well diode to form the ‘signal’ [16]. Monte Carlo simulations, using programs developed in-house and based on the software originally written by Joy [17], were used to predict electron trajectories in the sensor and the energy deposited along the tracks [13].

**Fig. 2:**
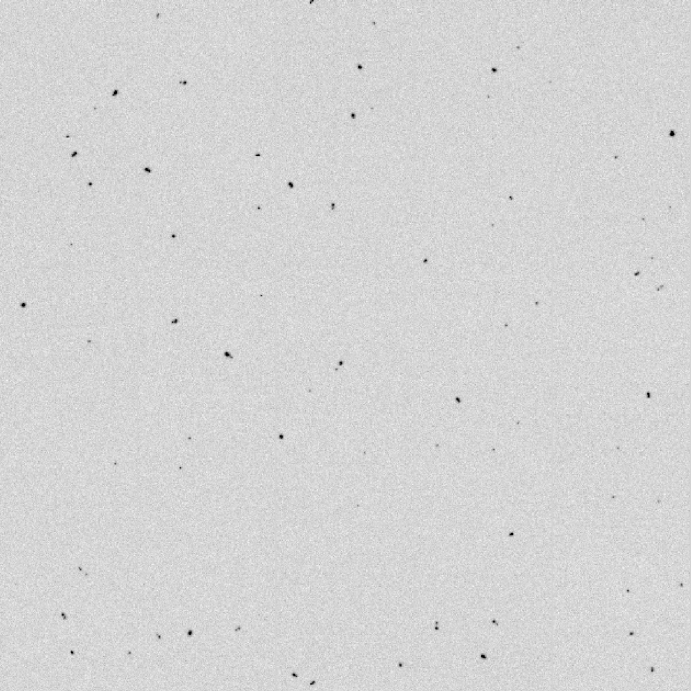
Some experimental images where the low incident electron flux allows individual electron events to be observed. This is a composite image consisting of 25 128×128 frames that have been juxtaposed, each frame showing between 1 and 5 events. The electron dose (∼1 electron/5000 pixels/frame) was kept at such a low level that the probability of two adjacent electrons was extremely small. Many of the events consist of two adjacent energy deposits, which arise from the incident and backscattered trajectories of those electrons that suffer backscattering. Electrons passing in only one direction and not suffering any backscattering appear as single events usually with a relatively small amount of energy deposited.

**Fig. 3:**
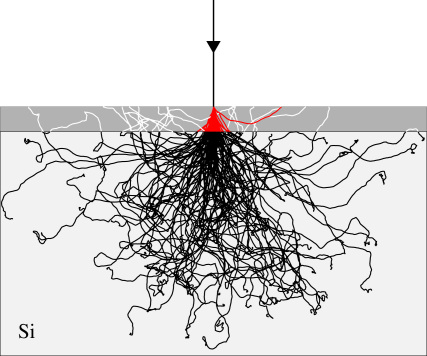
Monte Carlo simulation of 300 keV electron tracks in silicon. After backthinning to 35 μm, only those parts of the electron tracks highlighted in red would contribute to the recorded signal, which therefore is expected to have a much improved MTF. Before backthinning, the additional white tracks would contribute a low-resolution component to the signal together with contributions to the noise at both low and high spatial frequencies. The overall thickness of the silicon in the figure is 350 μm with the 35 μm layer that remains after backthinning shown in grey. The black electron tracks in the substrate deposit energy but contribute minimally to the measured signal.

The two main causes of poor resolution (MTF) are multiple scattering in the epilayer and backscattering in the substrate. Further, the signal electrons are spread out due to diffusion in the epilayer. In the case of a 5 μm passivation layer and 4 μm epilayer, the lateral diffusion can be described by a Gaussian with (1/e) value of 8.1 μm [13]. Similar values for diffusion have been reported by Battaglia et al. [18] at 200 keV for 10-μm-square pixels with a ∼10 μm epilayer. The track of a typical electron that undergoes backscattering is illustrated in Fig. 1—the incident electron traverses the epilayer initially in a forward direction and, after backscattering, a second time in the reverse direction. This can be observed in a non-backthinned detector (Fig. 2) where the two segments of the electron path are seen as closely spaced pairs of peaks. Fig. 2 is a composite image formed from 25 juxtaposed frames of 128×128 pixels. Each 128×128 frame shows 1–5 events but many frames had no events. This was achieved experimentally by reducing the electron dose to ∼1 incident electron/5000 pixels/frame. The probability of recording two closely spaced independent events is therefore extremely small. A Monte Carlo simulation is shown in Fig. 3 comparing tracks in the epilayer (white) solely due to backscattering with those that would occur whether or not the sensor was backthinned (red). To make matters worse, because the electron has slowed down due to the interactions in the substrate, the second traversal through the epilayer leads to a greater energy deposition.

## 3 Results and discussion

**Fig. 4:**
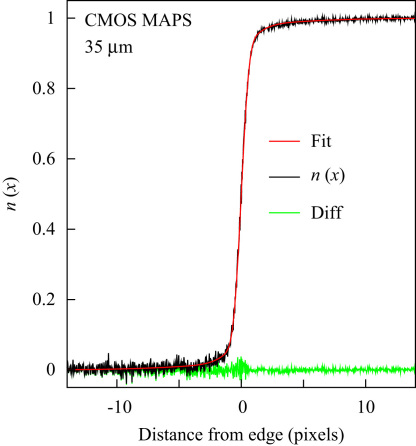
Normalized intensity distribution as a function of distance, in pixels, from the knife edge shown for the 35-μm-thick detector. The experimental intensity is black, the fit is grey (red) and the difference between them, shown in light-grey, is essentially pure noise. The fitted curve is from a model of the line spread function based on a sum of two Gaussians.

The MTFs of the detectors with different thickness were measured using the edge spread method (Fig. 4) in which the detector records the shadow of a straight edge placed in a uniform electron beam [19]. The edge is oriented at a slight angle to the detector and the average pixel value calculated as a function of perpendicular distance from the edge. After normalizing intensities to range from zero to one on opposite sides of the edge, the MTF can then be calculated from the resulting normalized edge spread, by taking the derivative to obtain the line spread function, and then the Fourier transform to obtain MTF. Instead of taking the numerical derivative of a noisy function we prefer to fit the edge spread function to a trial functional form from which the derivative and Fourier transform can be obtained analytically. This process is illustrated in Fig. 4 where the measured edge spread, fitted function, and difference is shown for a 35-μm-thick detector. A number of models were tried but a model for the line spread function based on a sum of (three) Gaussians was found to have sufficient flexibility.

**Fig. 5:**
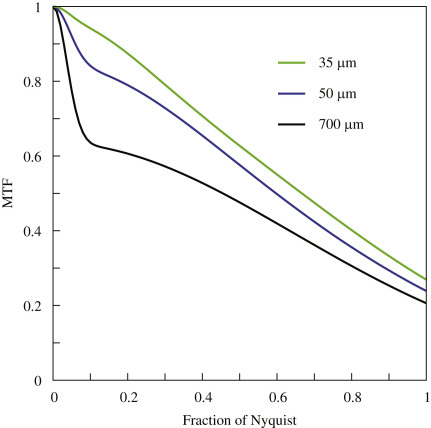
The experimentally determined MTF curves for 35 μm (dotted line, green), 50 μm (dashed line) and 700 μm (continuous line, black) thick detectors as a function of spatial frequency between zero and Nyquist frequency are shown. There is a substantial improvement in MTF as a result of backthinning, which is greatest for the 35 μm backthinned detector. From the size of the initial drop in MTF at low spatial frequency (<0.1 Nyquist), it appears that further backthinning down to the epilayer would produce only a small further improvement in MTF.

The effect of backthinning on the edge spread function is illustrated in Fig. 5 where the MTFs for detectors having thicknesses of 700, 50 and 35 μm are shown, and as expected, backthinning improves the MTF. The most dramatic effect of backthinning is to reduce the rapid fall off in the MTF at low spatial frequency and this leads to higher MTFs at all spatial frequencies. This increased MTF results in a greater proportional increase in DQE since DQE contains a MTF^2^ term. At this stage of detector development we do not attach too much importance to the exact values of DQE and MTF since these are dependant on factors such as pixel pitch, epilayer thickness, extent of doping, passivation process, diffusion lengths in the epilayer, etc. We conclude from this work that backthinning is an essential component of any desirable detector.
