# Supplementary figures and images for: Experimental observation of the improvement in MTF from backthinning a CMOS direct electron detector
Source: Ultramicroscopy. 2009 Aug;109(9-3):1144–7. doi: 10.1016/j.ultramic.2009.05.005 (PMC2937214; doi:10.1016/j.ultramic.2009.05.005)

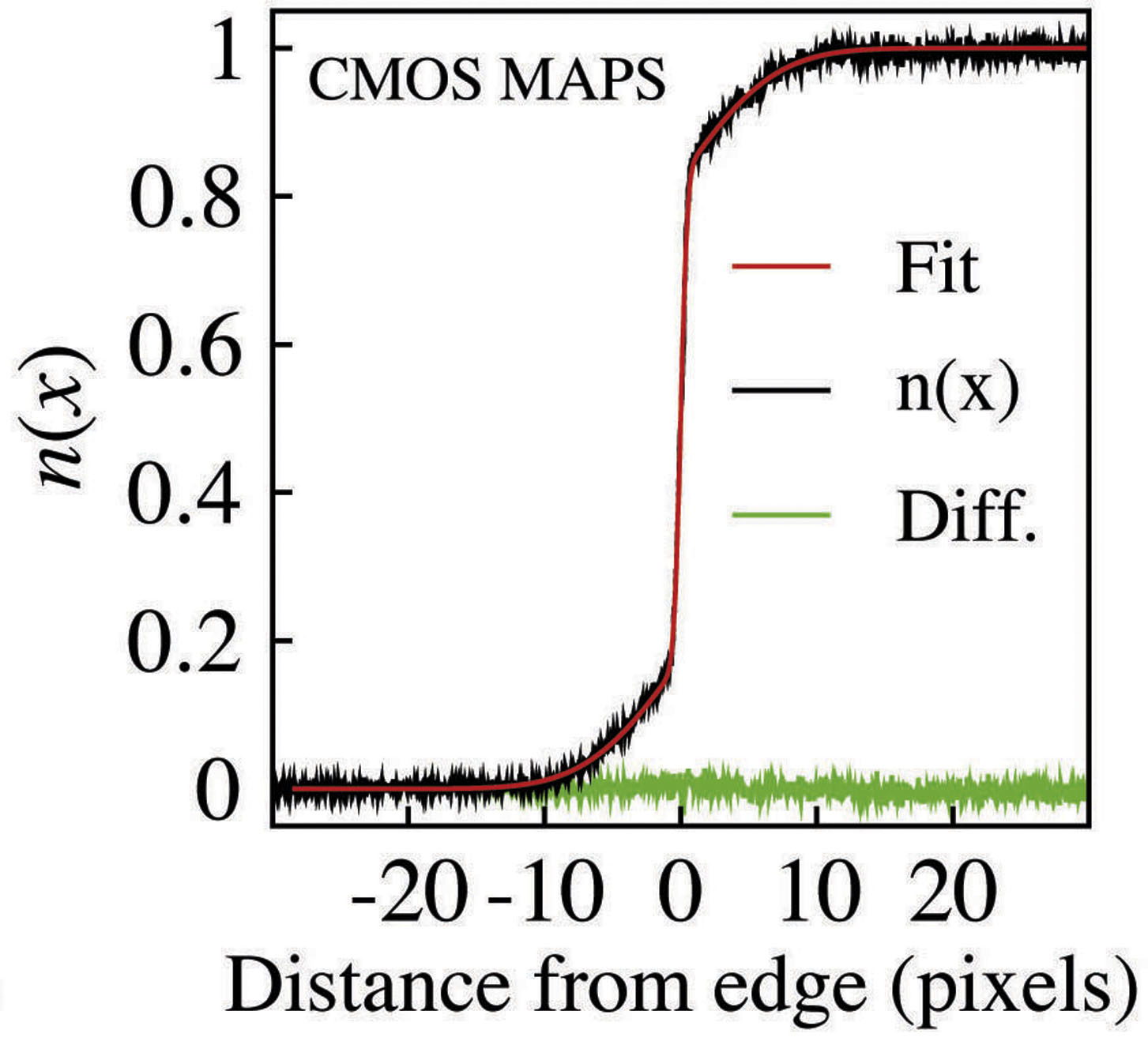

Supplement: Supplementary file 1 — Supplementary Figure [file mmc1.jpg]

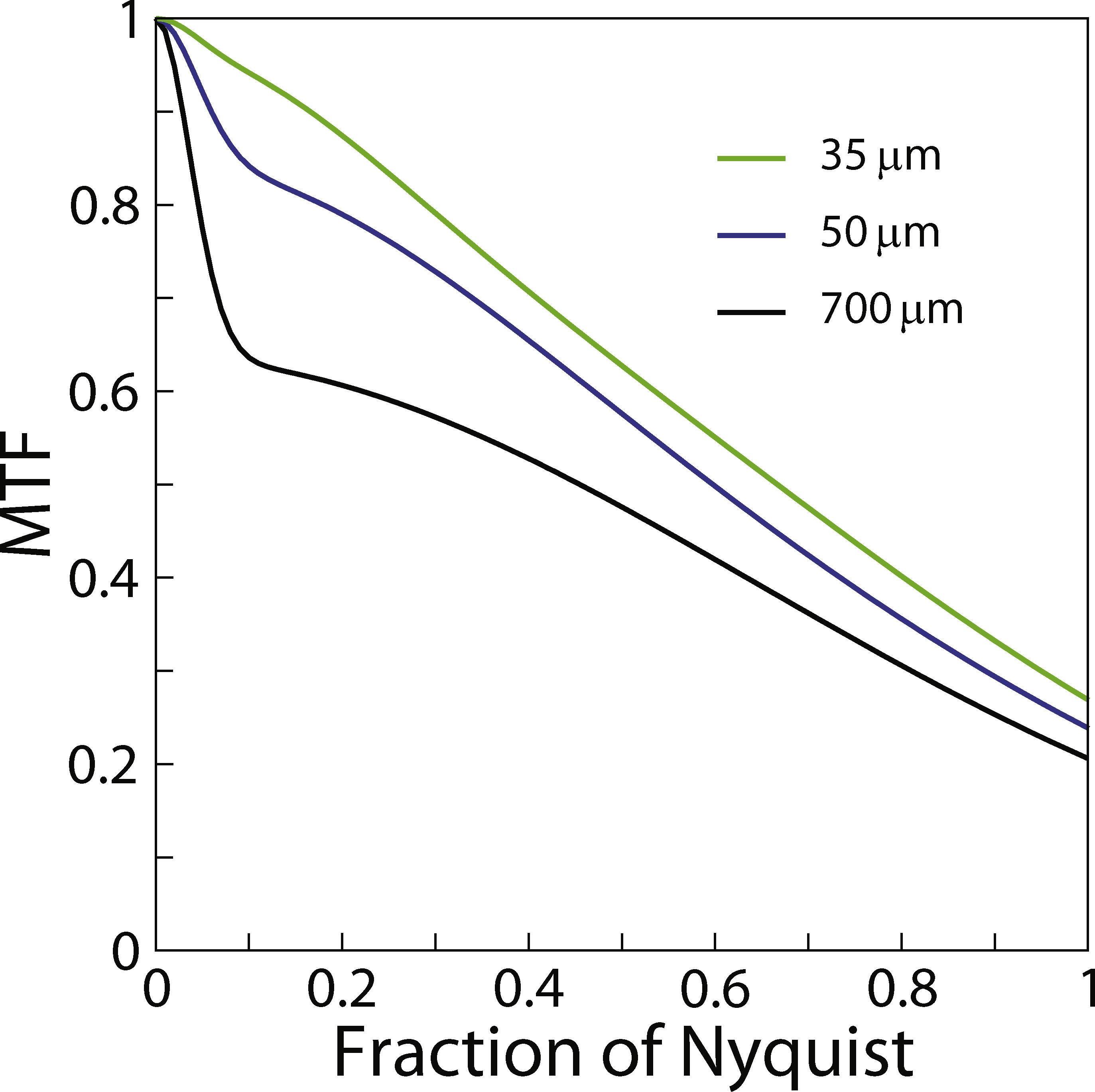

Supplement: Supplementary file 2 — Supplementary Figure [file mmc2.jpg]
